# NF-κB Immunity in the Brain Determines Fly Lifespan in Healthy Aging and Age-Related Neurodegeneration

**DOI:** 10.1016/j.celrep.2017.04.007

**Published:** 2017-04-25

**Authors:** Ilias Kounatidis, Stanislava Chtarbanova, Yang Cao, Margaret Hayne, Dhruv Jayanth, Barry Ganetzky, Petros Ligoxygakis

**Affiliations:** 1 Cell Biology, Development, and Genetics Laboratory, Department of Biochemistry, University of Oxford, South Park Road, Oxford OX1 3QU, UK; 2 Laboratory of Genetics, 425-G Henry Mall, University of Wisconsin, Madison, WI 53706-1580, USA

**Keywords:** *Drosophila*, NF-κB, Imd, Relish, brain, innate immunity, lifespan

## Abstract

During aging, innate immunity progresses to a chronically active state. However, what distinguishes those that “age well” from those developing age-related neurological conditions is unclear. We used *Drosophila* to explore the cost of immunity in the aging brain. We show that mutations in intracellular negative regulators of the IMD/NF-κB pathway predisposed flies to toxic levels of antimicrobial peptides, resulting in early locomotor defects, extensive neurodegeneration, and reduced lifespan. These phenotypes were rescued when immunity was suppressed in glia. In healthy flies, suppressing immunity in glial cells resulted in increased adipokinetic hormonal signaling with high nutrient levels in later life and an extension of active lifespan. Thus, when levels of IMD/NF-κB deviate from normal, two mechanisms are at play: lower levels derepress an immune-endocrine axis, which mobilizes nutrients, leading to lifespan extension, whereas higher levels increase antimicrobial peptides, causing neurodegeneration. Immunity in the fly brain is therefore a key lifespan determinant.

## Highlights

• Constitutive immunity predisposes to short lifespan with severe neurodegeneration
• Blocking constitutive immunity in glia rescues predisposed flies
• Suppression of immunity in glia of healthy flies triggers adipokinetic signaling
• This immune-endocrine axis mobilizes nutrients and extends active lifespan

## Graphical Abstract

**Figure:**
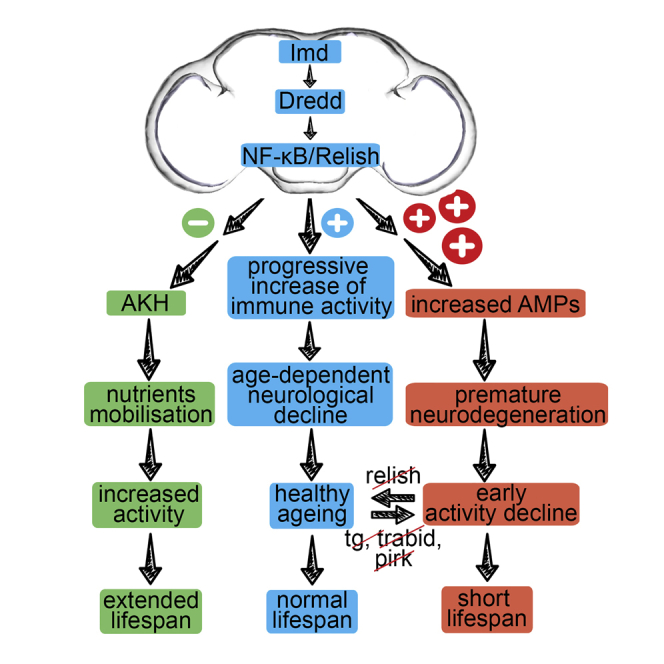

## Teaser

In humans, both healthy aging and age-dependent neurodegeneration are accompanied by an upregulation of innate immunity. What is the cause and what is the consequence remain unclear. Kounatidis et al. show that, in flies, NF-κB immune signaling controls lifespan in healthy flies, as well as in those predisposed to early neurodegeneration.

## Introduction

In the later stages of human life, there is a sustained increase in innate immune activity that has been associated with aging (Franceschi et al., 2007). This is in contrast to the induction of an immune response by infection and its rapid termination when the pathogen is cleared. This heightened age-associated pro-inflammatory activity has been termed “inflammageing” (Franceschi et al., 2007). It is thought to be partly balancing the exhaustion of T cell-mediated immunity caused by a decrease in the number of naive T cells produced by the thymus because of organism-wide cell senescence (Franceschi et al., 2007, Deeks, 2011). Other causes include the accumulation of tissue damage during the removal of senescent cells in various tissues and a defective autophagy response (Salminen et al., 2012). One of the most important transcriptional signatures in inflammageing of both humans and mice is the activation of the pro-inflammatory nuclear factor κB (NF-κB) pathway (de Magalhães et al., 2009, Zhang et al., 2013).

It has been hypothesized that, under certain conditions of heightened innate immunity, inflammageing may lead to age-associated brain neurodegeneration (Giunta et al., 2008). Although it is not known what predisposes individuals to disease, central to this immune-to-brain signaling are microglia. In the aging brain, the microglia have a primed phenotype as a consequence of changes in their local environment (Moreno et al., 2011). Nevertheless, whether the process involves the age-dependent activity of the NF-κB pathway is still an open question.

Taken together, the data mentioned above indicate the important influence of innate immunity on both healthy aging and age-related neurodegeneration. However, because cause and effect are so intricately linked (neurodegeneration itself causes inflammation), it is unclear how common themes in signaling may end up with such distinct phenotypes (healthy aging versus neurological disease). In this context, we used *Drosophila* to explore the potential influence of NF-κB-controlled immune signaling in predisposition to age-related neurological disease as well as healthy aging.

In *Drosophila*, systemic infection triggers two NF-κB signaling pathways; namely, Toll and immune deficiency (IMD). The latter pathway has extensive similarities with the tumor necrosis factor receptor 1 (TNFR1) signaling cascade (Kounatidis and Ligoxygakis 2012). Upon infection, activation of IMD is triggered when fragments of peptidoglycan released by Gram-negative bacteria or Gram-positive bacilli bind the transmembrane peptidoglycan recognition protein (PGRP)-LC or the intracellular PGRP-LE (Kaneko et al., 2006). The signal is then transduced through a receptor-adaptor complex to the NF-κB homolog Relish (Rel). Specifically, IMD (a RIP1 homolog) associates with the Fas-associated death domain protein (FADD), which then recruits the caspase-8 homolog death related ced-3/Nedd2-like caspase (DREDD), which is activated by ubiquitylation (Meinander et al., 2012). DREDD cleaves IMD, thus unmasking a domain of interaction with the *Drosophila* inhibitor of apoptosis-2 (dIAP-2), which ubiquitinates and stabilizes IMD (Paquette et al., 2010). This creates a transient signaling platform for the recruitment of transforming growth factor β (TGF-β)-activating kinase 1 (TAK1) and its binding adaptor TAB2 (Fernando et al., 2014). The TAK1/TAB2 complex mediates phosphorylation of the IκB kinase (IKK) on one hand and Jun nuclear kinase (JNK) on the other (Silverman et al., 2003). In turn, IKK phosphorylates the N-terminal domain of Rel, whereas DREDD cleaves the C-terminal. N-terminal Rel is then free to move to the nucleus and regulate transcriptional targets, including induction of antimicrobial peptide (AMP) genes (Stoven et al., 2003). As the signal is transmitted from the cell surface to the nucleus, there is negative regulation at every step. There is inhibition of PGRP-LC signaling by the transmembrane PGRP-LF (Basbous et al., 2011), inhibition of the receptor-adaptor complex through Rudra/Pirk (Aggarwal et al., 2008), and blocking of the signaling flow by successive de-ubiquitination enzymes targeting IMD (dUSP36) (Thevenon et al., 2009), TAK1 (the A20 homolog Trabid) (Fernando et al., 2014), or IKK (the cylidromatosis disease homolog cylindromatosis [CYLD]) (Tsichritzis et al., 2007). Moreover, ubiquitin-mediated proteolysis depletes the pathway from DREDD (via Dnr1) (Guntermann et al., 2009), TAK1 (via plenty of SH3 [POSH]) (Tsuda et al., 2005), and Relish (via ring and YY1 binding protein [dRYBP]) (Aparicio et al., 2013), whereas transglutaminase (TG)-catalyzed protein-protein cross-linking prevents Relish from entering the nucleus (Shibata et al., 2013). Finally, Caspar inhibits DREDD-dependent cleavage of Relish (Kim et al., 2006). In addition, there are extracellular negative regulators represented by secreted catalytic PGRP proteins (PGRP-LB and PGRP-SC), which reduce the epithelial and/or systemic response by scavenging peptidoglycan (Paredes et al., 2011). The safeguarding of the IMD pathway at all levels and with multiple means underlines the notion of an important cost paid if these safeguards were to decrease or collapse. Indeed, lack of Trabid, Pirk, PGRP-SC, or PGRP-LB compromise lifespan (Fernando et al., 2014, Paredes et al., 2011), whereas mutations in *dnr-1* or overexpression of AMPs in the brain result in neurodegeneration (Cao et al., 2013). Similarly, continuous overexpression of PGRP-LE, leading to chronic upregulation of AMPs, compromised lifespan in a Relish-dependent manner, linking immunity, inflammation, and longevity in flies (Libert et al., 2006). Moreover, TG has been associated with neurotoxicity in a spinocerebellar ataxia model (Lin et al., 2015),whereas mutations in *relish* suppress neurodegeneration in an ataxia-telangiectasia model (Petersen et al., 2013). Nevertheless, innate immune genes are upregulated in fly models of neurodegeneration, raising the possibility that this upregulation may be protective (Cantera and Barrio, 2015). In this context also, cause and consequence might be intimately linked.

In addition to the link between components of the immune system and neurodegeneration, there is an intimate connection between immunity and metabolism. In mammals, adipose tissues and infiltrating immune cells produce numerous bioactive factors that have pro-inflammatory or anti-inflammatory activities. Dysregulated production of so-called adipokines can contribute to the pathogenesis of obesity-linked metabolic disease (for a review, see Ouchi et al., 2011). These players have been shown to drive type 2 diabetes, whereas cytokines regulate lipid stores (for a review, see Donath and Shoelson, 2011). In flies, prolonged immune activation in the context of bacterial or viral infections has also been associated with deregulation of metabolism, mainly through the insulin signaling pathway (Dionne et al., 2006; DiAngelo et al., 2009). More recently, a switch between immunity and metabolism has been identified in the transcription factor Mef2. There, sustained immune activity removed Mef2 from metabolic regulation, whereas, in the absence of infection, Mef2 associated primarily with metabolic transcriptional signatures (Clark et al., 2013).

The results presented here show that, with age, NF-κB-dependent AMP gene expression increased, and this was accompanied by progressive neurodegeneration and locomotion decline. Constitutive NF-κB immune signaling (in *pirk*, *trbd*, or *tg* mutants) resulted in high head and brain AMPs. Flies had a short lifespan, severe neurodegeneration, and locomotor defects. Conversely, reducing the normal levels of NF-κB in the brain of healthy flies resulted in an extended lifespan with improved activity in old age, accompanied by increased hormonal signaling and elevated glucose, trehalose, and triglycerides. Our results demonstrate that IMD/NF-κB/Relish immune signaling in glia determines lifespan.

## Results

### Age-Dependent Immune Regulation in Flies

**Figure 1:**
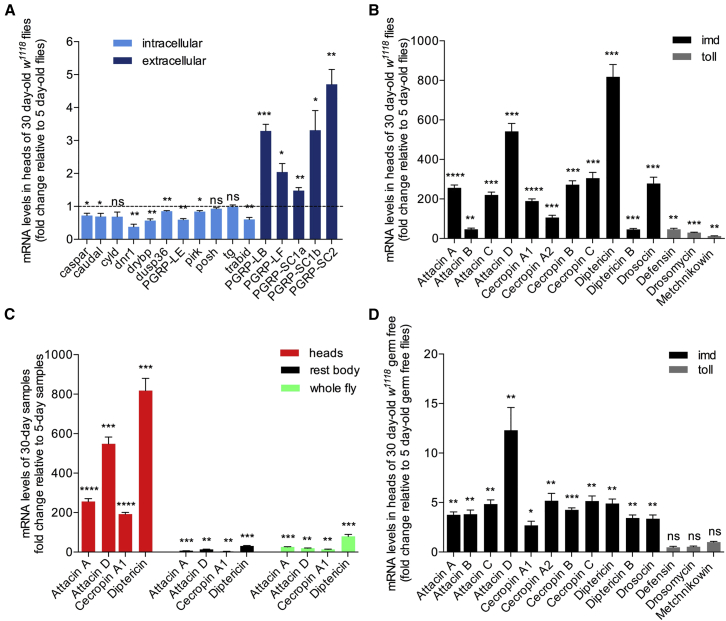
Healthy Aging in *Drosophila* Is Characterized by Age-Dependent Upregulation of Immunity (A–D) In the heads of 30-day old flies, gene expression of intracellular negative regulators of IMD was reduced, whereas gene expression of extracellular negative regulators was increased (A). This pattern was accompanied by a significant increase in AMP gene expression in the head (B), the rest of the body, and whole flies (C, comparative graph). IMD-dependent (but not TOLL-dependent) AMP gene expression was also increased with age in the heads of 30-day-old germ-free flies (D). Values shown are mean ± SEM.

We monitored the age-related expression of negative regulators of IMD, Toll, and JNK by comparing healthy whole flies, heads, and the rest of the body in the absence of infection. Our results indicated a consistent pattern: age-dependent gene expression of IMD *intracellular* negative regulators was significantly reduced in heads (Figure 1A) compared with the rest of the body (where expression of some was increased, compare Figure S1A with Figure 1A) or to whole flies (where expression appeared unchanged, compare Figure S1B with Figure 1A). In contrast, expression of IMD *extracellular* negative regulators was increased with age in heads (Figure 1A), the rest of the body (Figure S1A), and whole flies (Figure S1B). The decrease of intracellular negative regulation in the heads of aging flies was paralleled by a dramatic increase in AMP gene expression in the heads of 30- and 50-day-old flies (compare Figure 1B with Figures S1C and S1D for the rest of the body and whole flies respectively; see Figure 1C for a combined graph for a 30-day-old dataset; see Figure S1E for a 50 day-old dataset). We also observed a statistically significant increase in expression levels of *rel* in heads and whole bodies of 30-day-old flies compared with young 5-day-old controls (Figure S1F). *Dif* expression was also increased in the heads of 30-day-old flies (Figure S1F). However, this increase was not to the same extent as *rel* (3.2-fold versus 1.9-fold induction for *rel* and *dif*, respectively; Figure S1F). No significant change in expression of other components of the Toll or JNK pathway was observed with age (Figure S1F).

Comparison of AMP gene expression levels in the heads of conventionally reared versus germ-free flies indicated the importance of the microbial environment in the age-dependent increase observed (compare Figure 1D with Figure 1C). The largest part of this increase (>90%) was microbiota-dependent (Figures S1G and S1H). Nevertheless, this still indicated that there was a microbially independent, intrinsic propensity for age-dependent deregulation of the IMD pathway (Figures S1G and S1H). (Of note, that measurements under axenic conditions showed that the AMP gene expression increase concerned only IMD-regulated AMPs and not TOLL-dependent ones; Figure 1D).

**Figure 2:**
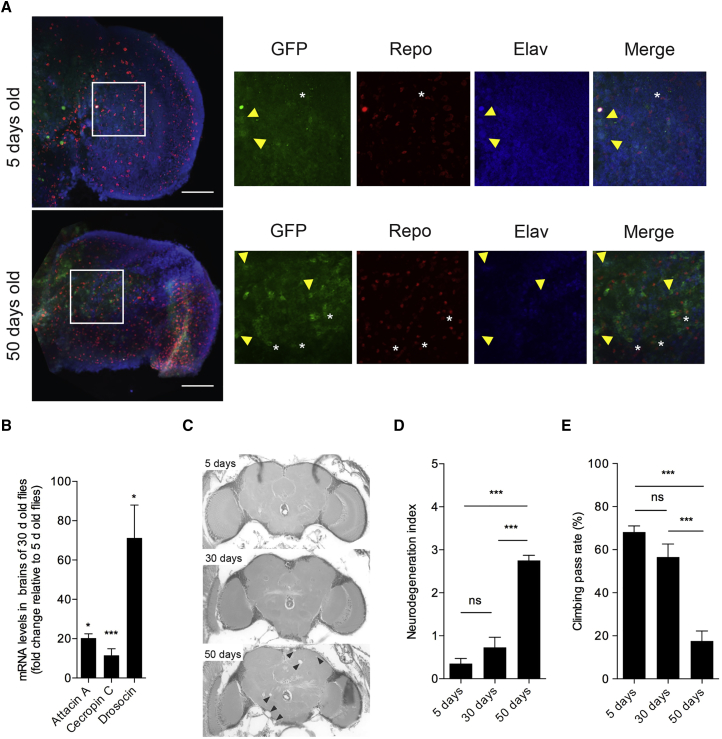
Increase of AMP Levels in the Brain Correlates with Neurological Decline (A) Confocal stacks of 5- and 50-day-old (top and bottom panels, respectively) *Attacin-GFP* optic lobes of brains immunostained for GFP (green), the glial marker Repo (red), and the neuronal marker Elav (blue). All three markers are shown in the merged image. An increase in GFP staining is observed in the brains of older flies, where GFP co-localizes with both Repo (asterisks) and Elav (arrowheads). The boxed regions show an enlarged image of the optic lobes. Representative images are shown. n = 19 (5 days old) and n = 12 (50 days old). Scale bars, 50 μm. (B–E) Significant age-dependent upregulation of AMPs in brains was also observed by qPCR (B). This increase was accompanied by neurodegeneration (arrowheads) in 50-day-old flies as observed (C) and quantified (D) in midbrain sections. This was coupled to a reduction in locomotor activity in 50-day-old flies (E). In all experiments, the fly strain used was *w*^*1118*^. Values shown are mean ± SEM. Asterisks denote statistically significant differences (^∗^p ≤ 0.05, ^∗∗∗^p ≤ 0.001. ns, non-significant.

We also observed an age-dependent increase in GFP expression in the brains of flies carrying a reporter gene in which *GFP* expression was under the control of the *attacinA* promoter (Tzou et al., 2000) (*Attacin-GFP*; Figure 2A). In addition, measurements by qPCR revealed an increase in AMP expression in brains of 30-day-old flies compared with their 5-day-old counterparts (Figure 2B). The results from these experiments indicated that the age-dependent increase in IMD-related AMP gene expression was concerning the brain itself and not just the surrounding tissues.

This age-dependent shift in IMD-related AMP transcription was the result of a specific pattern: a combined decrease in some negative *intracellular* regulators and an increase in *extracellular* negative regulators with a sum total culminating in an increase in *rel* mRNA levels and a rise in AMPs controlled by Rel. Furthermore, the age-dependent increase of AMPs was accompanied by an increase in brain neurodegeneration (see Figure 2C for sections, Figure 2D for index, and Table S1 for statistics) and locomotion defects (see Figure 2E and Table S2 for statistics) in 50-day-old flies. Taken together, the data above indicated a phenotype where an age-related increase in IMD/NF-κB-driven immunity was combined with a neurological decline. Whether this was a cause-and-effect relationship was our next question.

### Loss of NF-κB Intracellular Negative Regulation Predisposes to Early Neurodegeneration and Short Lifespan

**Figure 3:**
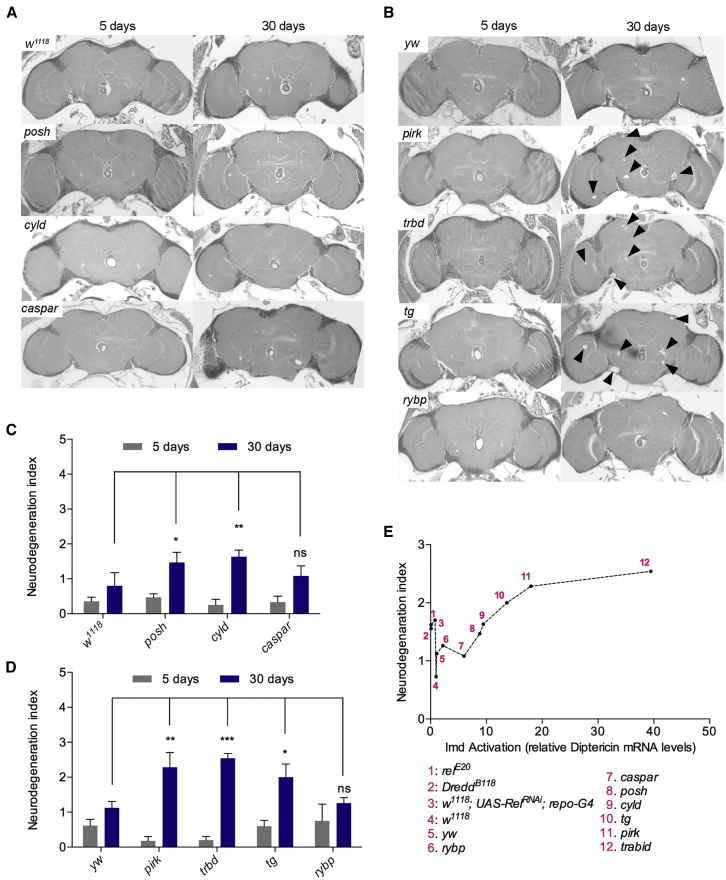
Loss of Intracellular Negative Regulators of NF-κB/Relish Triggers Early Onset of Neurodegeneration 5- and 30-day-old mutants for intracellular negative regulators of the IMD pathway, aged at 25°C, were examined for age-dependent neurodegeneration by histology and then quantified. (A) Midbrain sections of *caspar* mutant flies did not show any differences compared with their genetic background (age-matched w^*1118*^ controls). However, some sections showed an increase in *cyld* and *posh* mutants (data not shown). (B) Mutants for the intracellular negative regulators *pirk*, *trbd*, and *tg*, but not *rybp*, showed increased age-dependent neurodegeneration in midbrain sections. (C) When quantified, *caspar* mutant flies had a neurodegeneration index indistinguishable to *w*^*1118*^ controls, whereas *posh* and *cyld* had a significant increase in neurodegeneration. (D) Quantification of *pirk*, *trbd*, and *tg*, but not *rybp*, revealed a neurodegeneration index that was significantly different from *yw* controls. (E) Neurodegeneration index in correlation with Imd pathway output (as measured by *dipt* gene expression). The increase in neurodegeneration correlated with an increase in mRNA levels of *dipt*. Values shown are mean ± SEM. Asterisks denote statistically significant differences (^∗^p ≤ 0.05, ^∗∗^p ≤ 0.01, ^∗∗∗^p ≤ 0.001).

We reasoned that one way to explore this question would be to manipulate IMD/NF-κB signaling and measure any changes in the age-dependent neurological state of our flies. The causative relationship between increased IMD/NF-κB signaling levels and age-dependent neurological deterioration was identified previously for loss of Dnr1 (Cao et al., 2013). Therefore, we asked whether disabling other negative regulators and, thus, increasing IMD/NF-κB signaling levels could predispose flies to early onset of neurological decline, brain neurodegeneration, and, finally, reduced lifespan. Brain sections of 5- and 20- or 30-day-old loss-of-function mutants of the intracellular negative regulators (regardless whether their age-dependent expression declined or stayed the same) *posh*, *cyld*, and *caspar* (with their *w*^*1118*^ genetic background as a control) and *tg*, *trbd*, *pirk*, and *drybp* (with their *yw* genetic background as a control) as well as of the extracellular negative regulator *pgrp-lb* (with its *w*^*1118*^ genetic background as a control) were examined at both 25°C (see Figures 3A and 3B for brain sections and Figures 3C and 3D and Table S1 for index) and 29°C (accelerated aging; see Figures S2A, S2B, and S2E for brain sections, Figures S2C, S2D, and S2F for index, and Table S1 for statistical analysis). Early studies examining *Drosophila* lifespan at different temperatures have established a positive relationship between mortality and increase in temperature, therefore suggesting that flies are living and aging faster at 29°C than at 25°C (Alpatov and Pearl, 1929, Miquel et al., 1976). At both temperatures, our results revealed a statistically significant increase in the neurodegeneration index for three mutants in intracellular regulators of the IMD pathway; namely, *tg*, *trbd*, and *pirk*. In addition, plotting the neurodegeneration index against Imd transcriptional output (measured by gene expression levels of the AMP *diptericin*) indicated that the *dipt* levels in these three mutants correlated with the highest index (Figure 3E).

Of note, we found that the neurodegeneration index for conventionally reared mutants of PGRP-LB was comparable with the wild-type. This indicated that, despite the reduction in lifespan reported in the literature (Paredes et al., 2011), neurodegeneration was not the cause of early death in this mutant. Moreover, in contrast to *trbd* and *tg* (see below), under germ-free conditions, the lifespan of flies lacking PGRP-LB was restored to normal (Paredes et al., 2011 and data not shown). This result confirmed previous studies that indicated that the reduction in lifespan in PGRP-LB mutants was due to commensal dysbiosis (Paredes et al., 2011).

**Figure 4:**
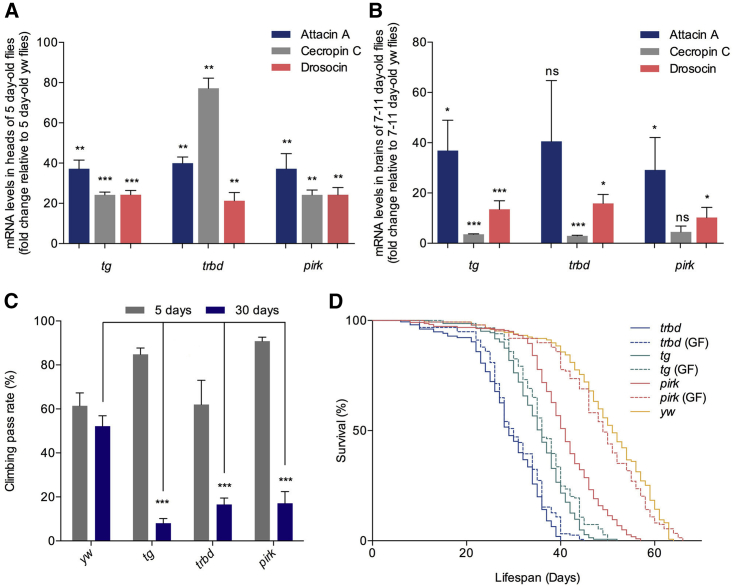
Loss of Intracellular Negative Regulators of NF-κB/Relish Leads to a Brain-Specific Increase in Expression of AMPs, Locomotor Defects, and Shorter Lifespan (A–D) A significant increase in AMP gene expression in comparison with *yw* controls was observed in heads (A) and dissected brains (B) of *pirk*, *trbd*, and *tg* mutants that correlated with a decrease in locomotor activity (C). The shorter lifespan of *pirk*, but not *trbd* or *tg* mutants, was rescued under germ-free (GF) conditions (D). Values shown are mean ± SEM. Asterisks denote statistically significant differences (^∗^p ≤ 0.05, ^∗∗^p ≤ 0.01, ^∗∗∗^p ≤ 0.001).

Along with the age-dependent neurodegeneration in *tg*, *trbd*, and *pirk*, these mutants showed a statistically significant increase in AMP gene expression in heads (Figure 4A), dissected brains (Figure 4B), and the rest of the body (Figure S3A) compared with the control. They were therefore exposed to “increased immune signaling from a young age” (predisposition) and were studied further. *Tg*, *trbd*, and *pirk* mutant flies exhibited an early reduction in locomotor activity (see Figure 4C and Table S2 for statistics) and showed a 24.2%, 37.1%, and 9.1% reduction in maximum lifespan, respectively, compared with wild-type controls (see Figure 4D and Table S3 for statistics). In the case of *trbd* and *tg*, lifespan patterns were not influenced by the absence of bacterial populations because they exhibited a statistically non-distinguishable reduction in lifespan under germ-free conditions (Figure 4D; Table S3). However, the reduction in lifespan of *pirk* mutants was rescued in germ-free flies (Figure 4D; Table S3), as has been reported elsewhere (Paredes et al., 2011). This result was in accordance with the published observation that *pirk* expression was regulated by the presence of the gut microflora (Lhocine et al., 2008) and underscored the differences between microbe-independent and microbe-dependent intracellular negative regulation of the IMD pathway (see Discussion).

In contrast to *pirk*, *trbd*, and *tg*, negative regulator mutants that did not show age-dependent neurodegeneration, such as *drybp* (Figure 3A) and *caspar* (Figure 3B), had levels of AMPs in their brains similar to controls (Figure S3B) and a lifespan that was not reduced compared with controls (the lifespan for *caspar* was actually increased; see Figure S3C and Table S3 for statistics). These results indicated that loss of different intracellular negative regulators had different effects on brain-specific AMP expression and, as a consequence, different effects on brain tissue, locomotion, and, ultimately, lifespan. This result also suggested possible tissue specificity (see Discussion). These differences notwithstanding, our results indicated that an increase in AMP gene expression did correlate with an increase in neurological decline and reduced lifespan. Indeed, Cao et al. (2013) have shown that overexpression of single AMPs increased neurodegeneration). We explored further the underlying neurological and lifespan phenotype and found that individual overexpression of *drosocin*, *attacinC*, or *cecropinA1* in neurons resulted in reduced lifespan and age-dependent climbing ability (Figures S3D and S3E, respectively). Similarly, overexpression of *drosocin*, *attacinC*, or *cecropinA1* in glia resulted in reduced lifespan and age-dependent climbing ability (Figures S3F and S3G, respectively).

### Silencing NF-κB in the Brain of Predisposed Flies Restores Lifespan and Suppresses Neurodegeneration

**Figure 5:**
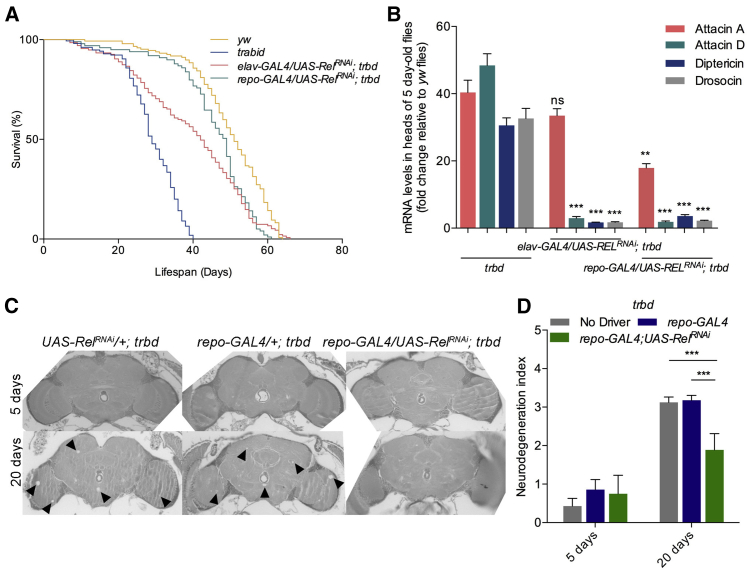
Predisposition to Neurodegeneration Is Suppressed by Reducing NF-κB/Relish in the Brain (A and B) In *trbd* mutants, reduction of lifespan (A) and upregulation of AMPs (B) were suppressed by silencing *rel* in neurons (*elav-GAL4*) or glia (*repo-GAL4)*. (C and D) Neurodegeneration in brain sections (C) and quantification (D) showed that lifespan extension was accompanied by fully rescued neurodegeneration only when *rel* was silenced in glia (compare with Figure S4B for sections when *rel* was silenced in neurons). Values shown are mean ± SEM. Asterisks denote statistically significant differences (^∗∗^p ≤ 0.01, ^∗∗∗^p ≤ 0.001).

**Figure 6:**
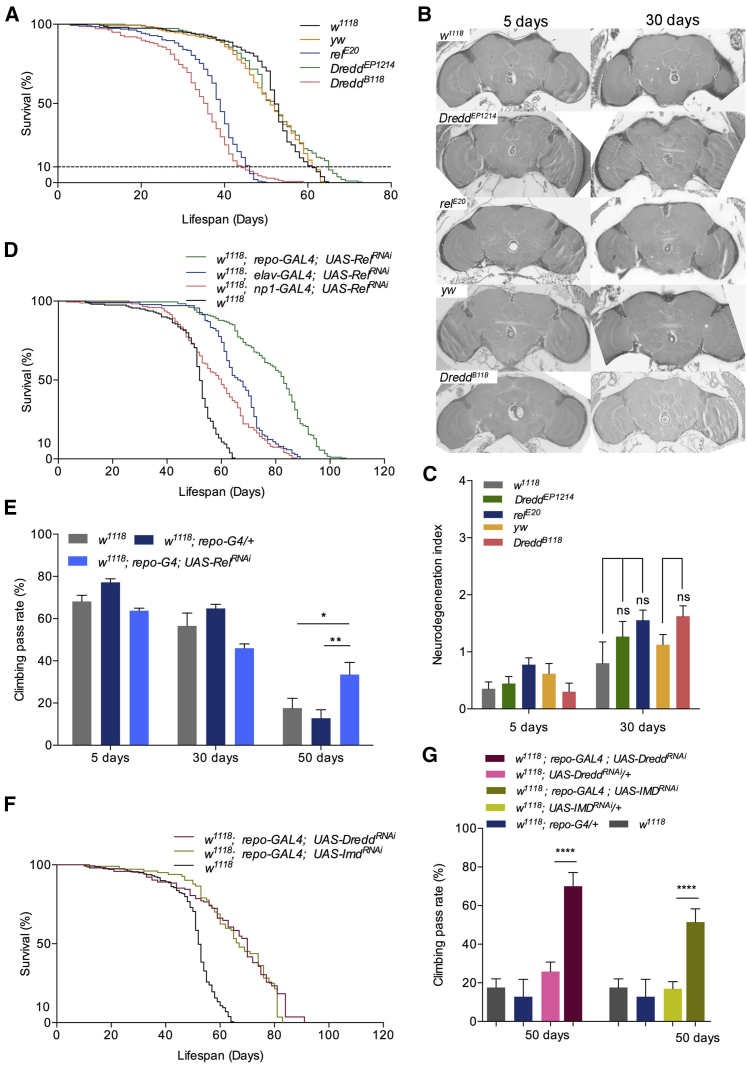
Glial Suppression of IMD/NF-κB Signaling in Healthy Flies Extends Active Lifespan (A–C) The maximum lifespan of a hypomorphic allele of *Dredd* (*Dredd*^*EP1214*^) showed a modest extension compared with *w*^*1118*^ controls. Nevertheless, midbrain sections (B) and quantification (C) of *yw; dredd*^*B118*^*, w*^*1118*^*; rel*^*E20*^-null mutants and a *w*^*1118*^*; dredd*^*EP1412*^ hypomorphic mutant showed no difference in neurodegeneration compared with controls. (D–G) RNAi-dependent silencing of *rel* in glia resulted in a very substantial extension of lifespan (D) and amelioration of the age-dependent decline in locomotor activity (E). Similarly, glial knockdown of *Dredd* and *Imd* resulted in lifespan extension (F) and amelioration of the age-dependent decline in locomotor activity (G). Values shown are mean ± SEM. Asterisks denote statistically significant differences (^∗^p ≤ 0.05, ^∗∗^p ≤ 0.01, ^∗∗∗∗^p ≤ 0.0001).

In *trbd* mutants, the median lifespan (LT_50_) was 29 days (Figure 5A; statistics in Table S3; see also Fernando et al., 2014). This phenotype was suppressed when *rel* was silenced in neurons (*w*^*1118*^*; elav-GAL4/UAS-Rel*^*RNAi*^*; trbd*, LT_50_ = 42) and even more when *rel* was suppressed in glia (*w*^*1118*^*; repo-GAL4/UAS-Rel*^*RNAi*^*; trbd*), with an LT_50_ of 48 days that was in line with the LT_50_ (52 days) of its genetic background (Figure 6A; Table S3). Mating rates were statistically indistinguishable in all different RNAi interventions compared with controls and *trbd* mutants (Table S6). Finally, the lifespan of *trbd* flies with only either of the GAL4 drivers or just the *UAS-Rel*^*RNAi*^ transgene was statistically indistinguishable from *trbd* alone (data not shown).

Compared with 5-day-old and 30-day-old controls, *rel-*silenced flies exhibited reduced AMPs in heads (Figure 5B). Neurodegeneration was reduced, and locomotor activity increased when *rel* was silenced in glia (see Figure 5C for brain sections, Figure 5D and Table S1 for index, and Figure S4A and Table S2 for locomotor assays), but not when it was silenced in neurons (see Figure S4B for brain sections, Figure S4C and Table S1 for index, and Figure S4D and Table S2 for locomotor assays). This result underlined the role of glia in the immunity-to-brain axis whereby suppressing immunity in glia rescued both neurodegeneration and locomotion and, thus, restored lifespan to wild-type levels. The data also supported the notion that predisposition to early neurodegeneration was determined by IMD/NF-κB pathway levels.

### Suppressing NF-κB Immune Signaling in Healthy Flies Extends Lifespan

If increased levels of IMD/NF-κB signaling led to the reduction of lifespan with early onset of age-related neurological decline, we wanted to test whether a decrease in normal IMD signaling levels in healthy flies from the beginning of their life could have the opposite effect. To this end, we initially assayed the effects on lifespan of two IMD-null mutants (*yw; dredd*^*B118*^ and *w*^*1118*^*; rel*^*E20*^), one hypomorphic mutant (*w*^*1118*^*; dredd*^*EP1412*^), and their genetic backgrounds to which the mutant strains were backcrossed for ten generations as recommended (He and Jasper, 2014). *Dredd*- or *rel*-null flies had a statistically significant reduction in lifespan compared with their genetic backgrounds (Figure 6A; Table S3). However, the hypomorphic *w*^*1118*^*; dredd*^*EP1412*^, although it showed the same significant reduction in LT_50_ as the null IMD pathway mutants compared with controls, also displayed a statistically significant increase in less than 90% (LT_90_) and maximum lifespan (Figure 6A; Table S3). However, no difference was observed in neurodegeneration in both brain sections (Figure 6B; Table S1) and index (Figure 6C; Table S4).

Nevertheless, this result suggested the possibility that a lower (but not null) IMD pathway activity could increase the maximum lifespan in some flies by reducing the cost of the age-dependent increase in levels of immune activity. To test this idea, we used the galactose responsive transcription factor 4/upstream activation sequence (GAL4/UAS) system to suppress *rel* via RNAi in either neurons (*elav-*GAL4) or glia (*repo-*GAL4) or the intestine (*np1-*GAL4). The latter GAL4 driver was used because the gut has been suggested to be the tissue where upregulation of *rel* causes age-related commensal dysbiosis (Guo et al., 2014). Previous studies have demonstrated that induction of the RNAi mechanism itself in the nervous system or gut does not influence adult lifespan (Alic et al., 2012). In addition, the mating rates were statistically indistinguishable in all different RNAi interventions compared with controls (Table S6).

Our results indicated a significant extension of lifespan when *rel* was suppressed in either neurons or glia (Figure 6D). In our hands (see Experimental Procedures), the genetic background *w*^*1118*^ had an LT_50_ of 52 days and an LT_90_ of 62 days, with a maximum lifespan of 65 days (see Figure 6D for a graph and Table S3 for statistics). In comparison, the *w*^*1118*^*; elav-GAL4; UAS-Rel*^*RNAi*^ LT_50_ was 67 days (an increase of 29%) and an LT_90_ of 79 days with a maximum lifespan of 88 days (35% increase). For *w*^*1118*^*; repo-GAL4; UAS-Rel*^*RNAi*^, the LT_50_ was 84 days (an increase of 61%), the LT_90_ was 95 days, and the maximum lifespan was 106 days (an increase of 63%; Figure 6D and Table S3). We also observed a comparatively modest (but statistically significant) increase in lifespan in *w*^*1118*^; *np1-GAL4; UAS-Rel*^*RNAi*^, as recently documented (Guo et al., 2014). In *w*^*1118*^; *np1-GAL4; UAS-Rel*^*RNAi*^ flies, the LT_50_ was 61 days and the maximum lifespan 70 days. However, *w*^*1118*^; *np1-GAL4* alone had a slightly increased LT_50_ compared with *w*^*1118*^, which was not the case for *w*^*1118*^; *elav-GAL4* or *w*^*1118*^; *repo-GAL4* (Figure 6D; Table S3). Interestingly, flies with glia- or neuron-specific suppression of immunity signaling did not show a reduction in brain neurodegeneration levels compared with controls (see Figure S5A for brain sections, Figure S5B for glia index, and Figure S5C for neuron index) even though AMPs in the heads were significantly reduced as expected (Figure S5D). This indicated that a reduction in the basal level of neurodegeneration was not the reason for lifespan extension. Nevertheless, glia-specific (but not neuron-specific) suppression resulted in increased locomotion in older flies (Figure 6E). This indicated that the lifespan extension in *w*^*1118*^*; repo-GAL4; UAS-Rel*^*RNAi*^ flies was not just an extension of the moribund phase but corresponded to more activity in old age. An extension of lifespan statistically comparable with *w*^*1118*^*; repo-GAL4; UAS-Rel*^*RNAi*^ flies was also observed in *w*^*1118*^*; repo-GAL4; UAS-dredd*^*RNAi*^ flies and *w*^*1118*^*; repo-GAL4; UAS-imd*^*RNAi*^ flies, two IMD pathway components upstream of Relish (see Figure 6F and Table S3 for statistics). To verify whether mating had an effect, we reproduced the same result in mated females only (see Figure S5E and Table S6 for statistics). Crucially, this extension was also correlated with a greater locomotor activity in later life (see Figure 6G and Table S5 for statistics). These results reinforced the notion that IMD/NF-κB signaling in the brain was linked to life expectancy in flies.

### An Immune-Neuroendocrine Axis Mediates Lifespan Extension in Flies

**Figure 7:**
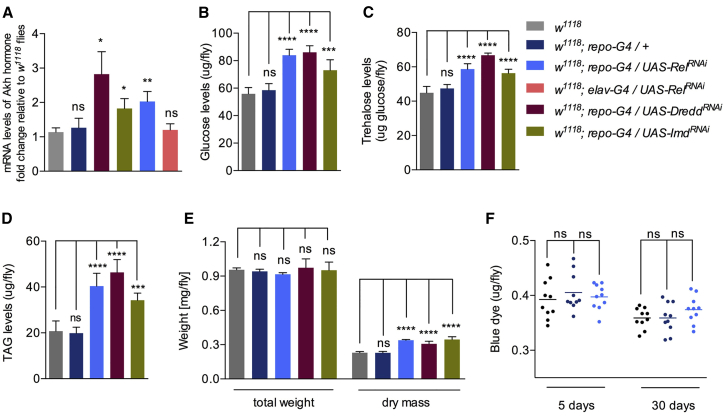
Lifespan Extension following Glial Suppression of IMD/NF-κB Signaling Is Accompanied by Metabolic Changes (A) The RNAi-dependent silencing of *rel*, *dredd*, and *imd* resulted in an increase in *Akh* transcription. (B–F) Increased AKH signaling triggered rises in (B) glucose, (C) trehalose, and (D) TAG levels that built up an increase in dry mass (E). Nevertheless, feeding rates were comparable with controls when *rel* was silenced in glia (F). Values shown are mean ± SEM.

The observed increase in physical performance in old age in flies with suppressed Imd signaling in glial cells was accompanied by doubling of the transcriptional levels of the adipokinetic hormone (Akh) hormone (Figure 7A). In contrast, this was not the case for mutants of negative regulators (*trbd*, *tg*, and *pirk*), where the pathway was constitutively active (Figure S6A).

Akh, which has glucagon-like functions, has been shown to increase the levels of blood glucose (Kim and Rulifson, 2004) as well as to extend lifespan and modulate climbing activity in *Drosophila* (Waterson et al., 2014). It has also been shown to sustain flight in locusts, tobacco hornworm moths, and certain beetles (reviewed in Gäde and Auerswald, 2003). In addition, its homolog in mice decelerates aging (Zhang et al., 2013). Consistent with the above, injections of a locust Akh in 30-day-old (see Figure 7B for a 5-cm climb in 10 s) and 50-day-old wild-type *Drosophila* increased locomotion (see Figure 7C for a 5-cm climb in 10 s and Figure 7D for a 2.5-cm climb in 10 s).

To corroborate the role of AKH as the effector of lifespan extension, we measured metabolic markers that have been associated with AKH signaling in *Drosophila* (Lee and Park, 2004, Bharucha et al., 2008). 30-day-old *w*^*1118*^*; repo-GAL4; UAS-Rel*^*RNAi*^ flies showed a statistically significant increase in glucose levels (free glucose levels in whole flies; Figure 7B) as well as in circulating trehalose (representing glucose dimers; Figure 7C). Of note is that levels of glycogen, which represented stored glucose, were unchanged relative to controls (Figure S6E). Consistent with the action of AKH signaling on mobilization of lipid stores, triglycerides (TAGs) as well as total dry mass (but not total weight) were increased in long-lived flies (Figure 7E). In contrast, total protein levels were indistinguishable from *w*^*1118*^ controls and *w*^*1118*^*; repo-GAL4* flies (Figure S6F). Finally, the metabolic changes observed were not due to calorie restriction because the feeding rates between long-lived and control flies were statistically indistinguishable (Figure 7F). Taken together, the above results indicated that, during healthy aging, suppression of NF-κB activity delayed neurological decline through a mechanism involving an immune-endocrine axis that seems to be evolutionary conserved in mice (Zhang et al., 2013).

## Discussion

### Loss of IMD Homeostasis Predispose to Age-Dependent Neurodegeneration

We have shown that, in *Drosophila*, an age-related pattern of increased NF-κB-controlled immune activity is caused by a parallel reduction in intracellular negative regulation. As seen in germ-free flies, this age-dependent rise in immune levels was observed downstream of IMD but not TOLL. Moreover, axenic conditions revealed that this increase was, to a large extent, due to the microbial environment. However, the trend of this increase (albeit at a reduced scale) remained in germ-free flies, indicating a mechanism independent of the microbiota.

This phenomenon was also accompanied by neurological and locomotor decline, but it was unclear whether this was a consequence of increased immune activation. If this were the case, we hypothesized that loss-of-function mutations in intracellular negative regulators of IMD would predispose flies to high AMP levels and, thus, to immune-dependent early neurodegeneration. Indeed, overexpression of AMPs with *repo-* or *elav-GAL4* can cause lesions in the brain at 25 days of age (Cao et al., 2013). Moreover, overexpression of the AMPs *drosocin*, *attacinC*, and *cecropinA1* in neurons or glia was accompanied by a reduction in lifespan and early appearance of locomotor defects in comparison with controls. Taken together, these results indicate a causative relationship between high levels of AMPs and neurodegeneration. Indeed, plotting the neurodegeneration index against the levels of the AMP gene *diptericin* in various mutant and control genetic backgrounds showed that increased neurodegeneration correlated with increased *dipt* levels (Figure 3E).

Nevertheless, Petersen et al. (2013) have suggested that it shows correlation, not causation, because *imd* alleles still show neurodegeneration. This view correlates with our results showing that both loss of function of IMD signaling components as well as silencing by RNAi in the brain of these same components did not reduce basal neurodegeneration. Therefore, the basal levels of neurodegeneration in healthy aging are primarily NF-κB-independent. Alternatively, the histological method we have used may not be sensitive enough to detect any potential small changes. Nevertheless, downregulation of NF-κB extends the active lifespan despite levels of neurodegeneration remaining constant. This shows that, when IMD is under normal regulatory conditions, longevity is determined by control of metabolism rather than neurodegeneration (see below). Conversely, when IMD control is lost, and AMPs increase to toxic levels, causing early neurodegeneration (Cao et al., 2013; this study), NF-κB activity is the dominant lifespan determinant over and above any metabolic process.

Loss of four intracellular IMD negative regulators, Trbd, Pirk, Tg (this study), and Dnr-1 (Cao et al., 2013), resulted in early neurodegeneration in combination with a reduction in lifespan. Nevertheless, the IMD pathway is negatively regulated at most steps. Therefore, the question arises of why loss of these particular four intracellular negative regulators produced age-dependent neurodegeneration.

Our view is that the data underscore the context and/or tissue-dependent nature of IMD signaling and the difference between intracellular control exerted when interacting with the gut microbiota and/or upon infection (*dusp36*, *pgrp-le*, *pirk*, and *drybp*) (see Thevenon et al., 2009, Bosco-Drayon et al., 2012, Lhocine et al., 2008; and Aparicio et al., 2013; respectively) versus infection-independent homeostatic control (*dnr-1*, *trbd*, and *tg*) (see Cao et al., 2013, Fernando et al., 2014; and Shibata et al., 2013; respectively). For example, Pirk is the upstream-most flora-dependent intracellular negative regulator that is a target of the pathway, and, therefore, its loss will result in the absence of an important negative feedback loop (Lhocine et al., 2008). Nevertheless, this feedback loop is not important for lifespan in the absence of the gut microflora (Paredes et al., 2011). In contrast, flies reared under germ-free conditions reveal that intracellular regulators such as Trbd are important for IMD pathway homeostasis independent of the microbial environment (Fernando et al., 2014). A non-canonical or tissue-specific IMD pathway may also be at play in the brain, as has been suggested for the fly retina (Chinchore et al., 2012) or in an ataxia telangiectasia model (Petersen et al., 2013).

Loss of negative regulation in IMD signaling in the brain led to tissue disruption and early neurodegeneration. This phenotype was accompanied by a severe reduction in lifespan and locomotor defects. However, when Relish was silenced in glia of *trbd* mutants, brain lesions, lifespan, and locomotion were restored and comparable with healthy control flies. This showed that all effects were due to the derepression of NF-kB signaling.

Our results indicate that reduction of lifespan in *tg* and *trbd* is microbe-independent. We suggest that the main reason for this lifespan reduction is the microbiota-independent part of the early increase in AMPs in those mutants. Interestingly however, the lifespan reduction in *pirk* mutant flies was rescued under germ-free conditions. Because *pirk* mutants display early neurodegeneration, this raises the possibility of a gut-brain axis that may include a microflora-dependent component, which, in turn, influences brain neurodegeneration. Indeed, it has been shown previously that, as flies age, their gut becomes leaky, resulting in aging decline with a strong microbiota-driven component (Rera et al., 2012). More work is needed to understand this interaction.

Finally, our work indicates that the age-dependent regulation of the Imd pathway is at a state of allostasis (when homeostasis is achieved through change). Taken at specific time points, signaling output is defined by the microbial environment, and, in the absence of infection, it is tightly regulated to a basal level. However, this basal level increases in an age-dependent manner. By culturing germ-free *Drosophila*, we were able to pinpoint the component of this signaling that is microbe-independent and intrinsic to the host.

### Suppression of IMD Extends Lifespan

Lowering the levels of IMD signaling by RNAi in glia of healthy flies extended the lifespan by more than 60% in LT_50_ and increased physical activity in old age. Our results agree with a previous observation that pharmacological inhibition of NF-κB modestly extends the *Drosophila* lifespan (20% of LT_50_; Moskalev and Shaposhnikov, 2011). However, we go further to indicate mechanism and tissue specificity. Lifespan extension was not achieved by lower levels of neurodegeneration but by an increase in AKH signaling. This indicated that the basal level of neurodegeneration in healthy flies was independent of IMD/NF-κB.

Extension of lifespan was accompanied by an altered metabolic profile, including statistically significant increases in free glucose, trehalose, and TAGs, whereas there was no significant difference in glycogen and protein levels. This suggested a shift toward catabolism, leading to the immediate use of carbohydrates and fats (as opposed to storing them) to fulfill the energetic requirements of long-lived flies. Increases in glucose, trehalose, and TAGs were consistent with the upregulation of *akh* expression and confirmed IMD signaling as a potent modulator of lifespan upstream of AKH.

More work is needed to identify the NF-κB targets in glia that enable AKH activation and establishment of this immune-neuroendocrine axis. We speculate that this is not a direct transcriptional relationship because *akh cis*-regulatory elements do not have NF-κB binding sites (unpublished data). One possible connection however, could be through Mef2 (Clark et al., 2013). Mef2 is an in vivo immune-metabolic switch controlled by NF-κB that can alternate between immune and metabolic gene expression. Upon infection, NF-κB activity recruits Mef2 as an immune regulator. A tempting hypothesis is that, by lowering NF-κB signaling under homeostatic conditions, Mef2 is freer to increase its involvement in metabolic activity.

Our results point to IMD/NF-κB signaling in the brain as a major determinant of *Drosophila* lifespan. The LT_50_ (84 days) of *w*^*1118*^*; repo-GAL4; UAS-Rel*^*RNAi*^ is a statistically indistinguishable percent extension of LT_50_ compared with *w*^*1118*^*; methuselah* (Lin et al., 1998), lifespan extension by loss of insulin signaling (Clancy et al., 2001), or calorie restriction (Mair et al., 2003). Our data put under an evolutionary perspective studies in mice where a similar NF-κB-controlled immune-neuroendocrine integration led to modulation of hormone levels and lifespan extension (Zhang et al., 2013). However, our results go further to indicate that NF-κB signaling levels regulate both healthy aging as well as age-related neurodegenerative disease. Given the evolutionary conservation of NF-κB innate immune signaling in flies, mice, and humans, opposing the inflammatory effects of NF-κB may represent a common strategy to increase active lifespan in both the context of healthy aging as well as in cases of predisposition to age-related neurological disease.

## Experimental Procedures

### *Drosophila* Stocks and Genetics

Flies were maintained on cornmeal-molasses medium at 25°C unless otherwise stated. For a complete list of the strains used, check the Supplemental Experimental Procedures.

### Lifespan Studies and Production and Maintenance of Germ-free Flies

For lifespan studies, all fly strains were backcrossed for ten generations to their respective genetic background as recommended previously (He and Jasper 2014). When these “lifespan-ready” strains were established, cohorts of 20 flies (10 males and 10 females) were put in vials and monitored for their survival in 12 biological replicates (n = 240/strain). The flies were transferred to fresh food every 2 days. Germ-free flies were produced as described previously (Fernando et al., 2014) and checked every 5 days for bacterial contamination by performing PCR analysis on fly homogenates using 16S eubacterial primers (63F//1387R) as well as by culturing the homogenates on LB plates.

### Histology and Neurodegeneration Score

Histological analysis and determination of the neurodegeneration index were done as described previously (Cao et al., 2013). For a detailed description, check the Supplemental Experimental Procedures.

### Gene Expression in Brains

Quantitative real-time PCR was used to measure mRNA expression. 30–40 fly brains were dissected at the indicated time points, and RNA was isolated using TrizolRT (Molecular Research Center) according to the manufacturer’s instructions. cDNA was prepared from 0.5 μg total RNA using the iScript cDNA synthesis kit (Bio-Rad). Real-time PCR was carried out using iQ SYBR Green Supermix (Bio-Rad). Primer sequences are presented in the Supplemental Experimental Procedures.

### Gene Expression in Heads, the Rest of the Body, and Whole Flies

Total RNA was extracted from whole flies (6 females), the rest of the body (8 females), and heads (30 females) using the Total RNA Purification Plus kit (Norgen Biotek), and cDNA was prepared from 0.5 μg total RNA using the Maxima First Strand cDNA synthesis kit (Thermo Scientific). Triplicate cDNA samples were amplified with the SensiFASAT SYBR No-ROX kit (Bioline) in a Corbet Rotor-Gene 6000 qPCR machine (QIAGEN) according to the manufacturer’s protocols. Primer sequences are presented in the Supplemental Experimental Procedures.

### Statistical Analysis

All statistical analyses were performed using GraphPad Prism software (GraphPad). All qPCR data were analyzed using nonparametric unpaired Student’s t test corrected for multiple comparisons (Bonferroni correction). Significant differences between the survival data of different genotypes were identified using log rank and Wilcoxon tests (chi-square and p values). The neurodegeneration index and climbing assay data were analyzed using one- or two-way ANOVA with Tukey and Bonferroni post tests, respectively. In all tests, p < 0.05 was considered significant.

## Author Contributions

P.L., I.K., and S.C. designed the experiments. I.K. and S.C. performed the experiments with input from Y.C., M.H., and D.J. I.K., S.C., and P.L. analyzed the data with input from B.G. P.L. wrote the paper with input from I.K., S.C., and B.G.

## Supplemental Information

Document S1. Supplemental Experimental Procedures and Figures S1–S6

Table S1. Genotypes and Quantification of Neurodegeneration in Each Figure, Related to Figures 2, 3, 5, and 6

Table S2. Genotypes and Quantification of Locomotor Activity in Each Figure, Related to Figures 2 and 4

Table S3. Statistical Analysis of Lifespan in the Respective Figures, Related to Figures 4, 5, and 6

Table S4. Statistical Analysis of Neurodegeneration in the Respective Figures, Related to Figure 6

Table S5. Statistical Analysis of Locomotor Activity in the Respective Figures, Related to Figure 6

Table S6. Courtship Index and Wing Extension Index, Related to the Results

Document S2. Article plus Supplemental Information
