# Supplementary material for: NF-κB Immunity in the Brain Determines Fly Lifespan in Healthy Aging and Age-Related Neurodegeneration
Source: Cell Rep. 2017 Apr 25;19(4):836–48. doi: 10.1016/j.celrep.2017.04.007 (PMC5413584; doi:10.1016/j.celrep.2017.04.007)
Supplement: Document S1. Supplemental Experimental Procedures and Figures S1–S6 [file mmc1.pdf]

Cell Reports, Volume *19*

Supplemental Information

NF-κB Immunity in the Brain Determines Fly Lifespan in Healthy Aging and Age-Related Neurodegeneration

Ilias Kounatidis, Stanislava Chtarbanova, Yang Cao, Margaret Hayne, Dhruv Jayanth, Barry Ganetzky, and Petros Ligoxygakis

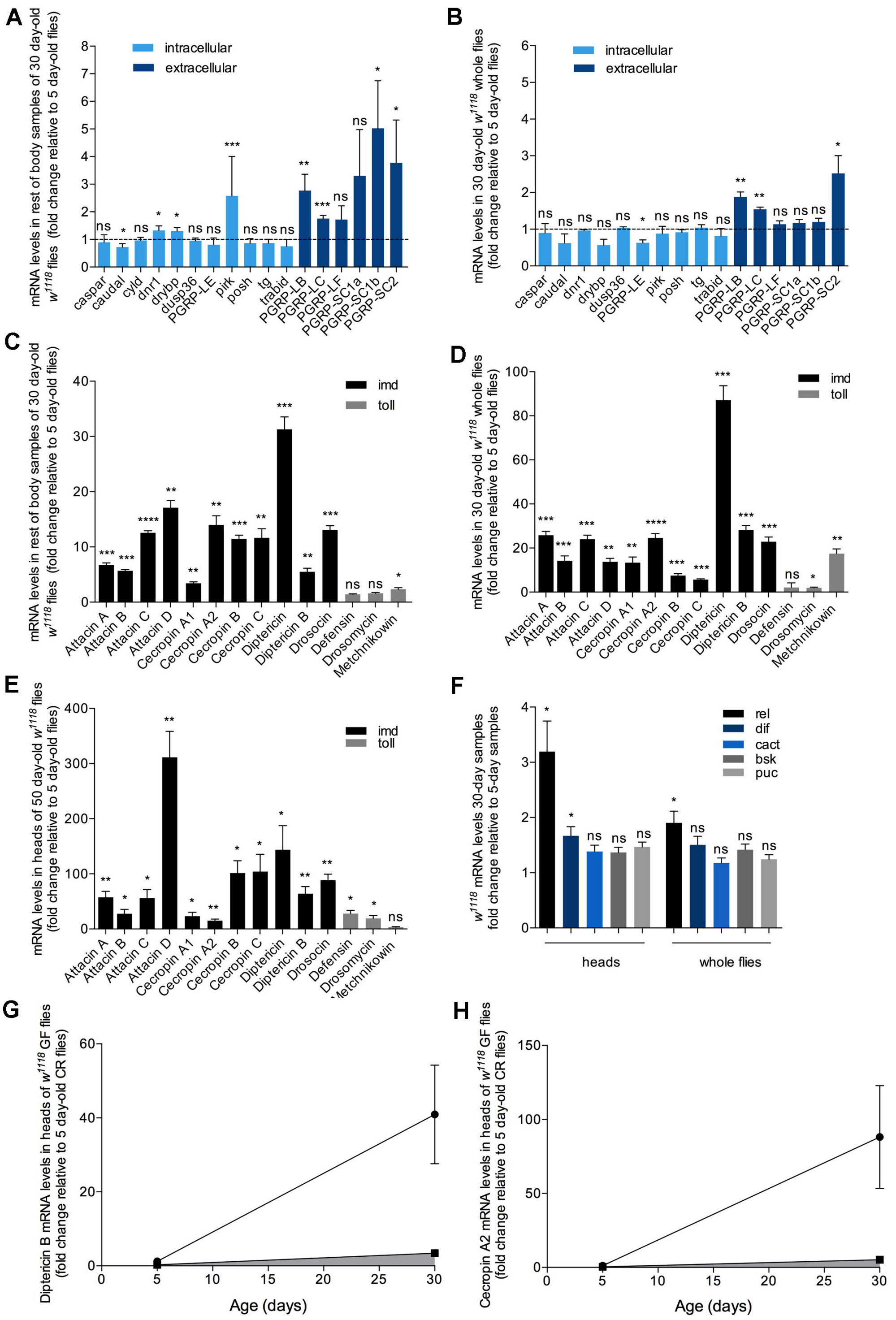

Figure S1 related to Figure 1.

Figure S1. Expression of immunity genes in conventionally reared flies. Gene expression of intracellular negative regulators of IMD stays unaltered in the rest of the body **(A)** and in whole flies **(B)** in 30-day old adults. In contrast, gene expression of extracellular negative regulators was increased in the rest of the body **(A)** and whole flies **(B)**. This pattern was accompanied by a large increase in AMP gene expression in rest of bodies **(C)** and whole flies **(D)**. **(E)** AMP gene expression levels in 50-day old w^{1118} fly heads. **(F)** Gene expression of *rel* as well as TOLL and JNK pathway components in 30-day old w^{1118} flies. Asterisks denote statistically significant differences (* $p \leq 0.05$; ** $p \leq 0.01$; *** $p \leq 0.001$; **** $p \leq 0.0001$; ns, non-significant). Values shown are mean ±SEM. To identify the component of AMP increase that was microbe-independent during aging we plotted Diptericin-B levels (G) and Cecropin A2 levels (H) during aging, in conventionally reared (CR) flies (solid cycles) and germ free (GF) flies (solid squares). Values shown are mean ±SEM.

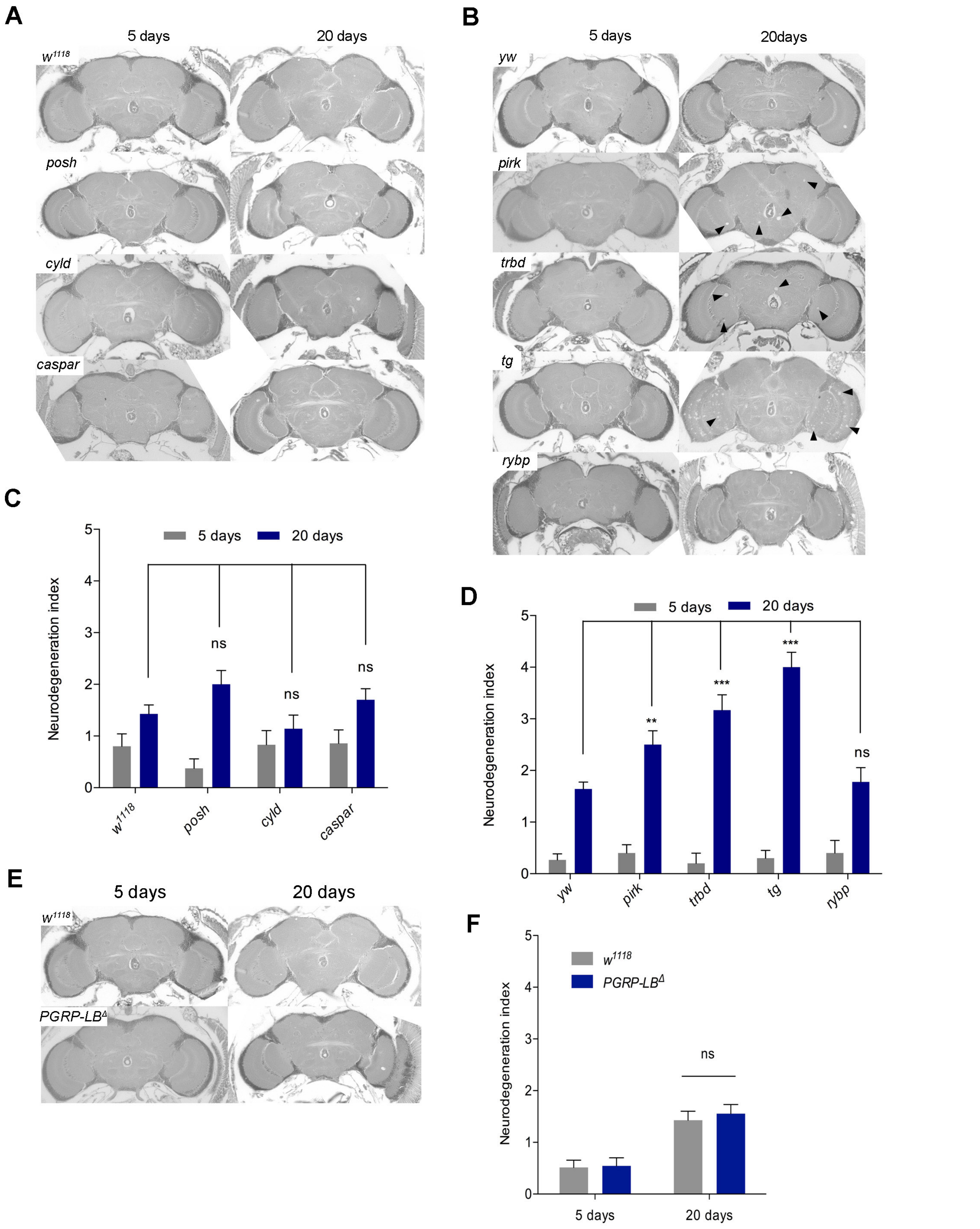

Figure S2 related to Figure 3.

Figure S2. Screening mutants of IMD pathway intracellular negative regulators for neurodegeneration in conditions of accelerated ageing (29°C). Several mutants for intracellular regulators were found to have age-dependent neurodegeneration [midbrain sections **(A, B)** and index **(C, D)**]. In contrast, neuropathology in mutants for the extracellular negative regulator PGRP-LB did not differ from controls [midbrain sections **(E)** and index **(F)**]. Values shown are mean ±SEM (* $p \leq 0.05$; *** $p \leq 0.001$; ns, non-significant).

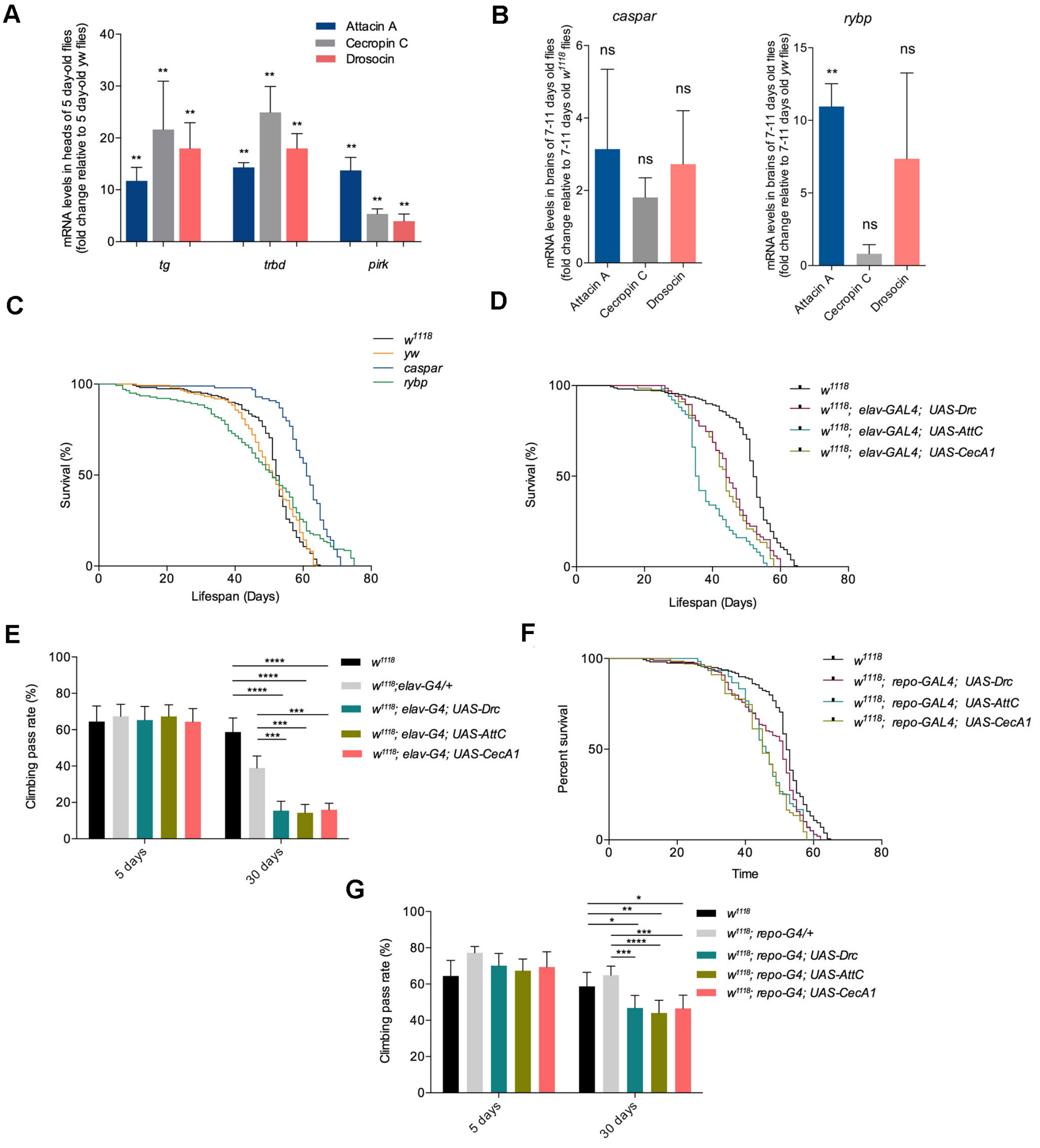

Figure S3 related to Figures 3 and 4.

Figure S3. AMPs expression in Imd negative regulator mutants, lifespan of *rybp* and *caspar* mutants and the effect of expressing individual AMPs on lifespan and locomotor activity. (A) Statistically significant increase in AMP gene expression was observed from an early age (5-day old flies) in *tg, trbd* and *pirk* mutants. In contrast, no important changes in brain AMPs gene expression **(B)**, nor reduction in lifespan **(C)** were observed for *rybp* and *caspar* mutants, which did not exhibit early onset neurodegeneration. Expressing individual AMPs in neurons significantly reduced lifespan **(D)** and locomotion **(E)**. Similarly, AMP expression in glia compromised lifespan **(F)** and locomotor activity **(G)**. Values shown are mean ±SEM (** $p \leq 0.01$; ns, non-significant).

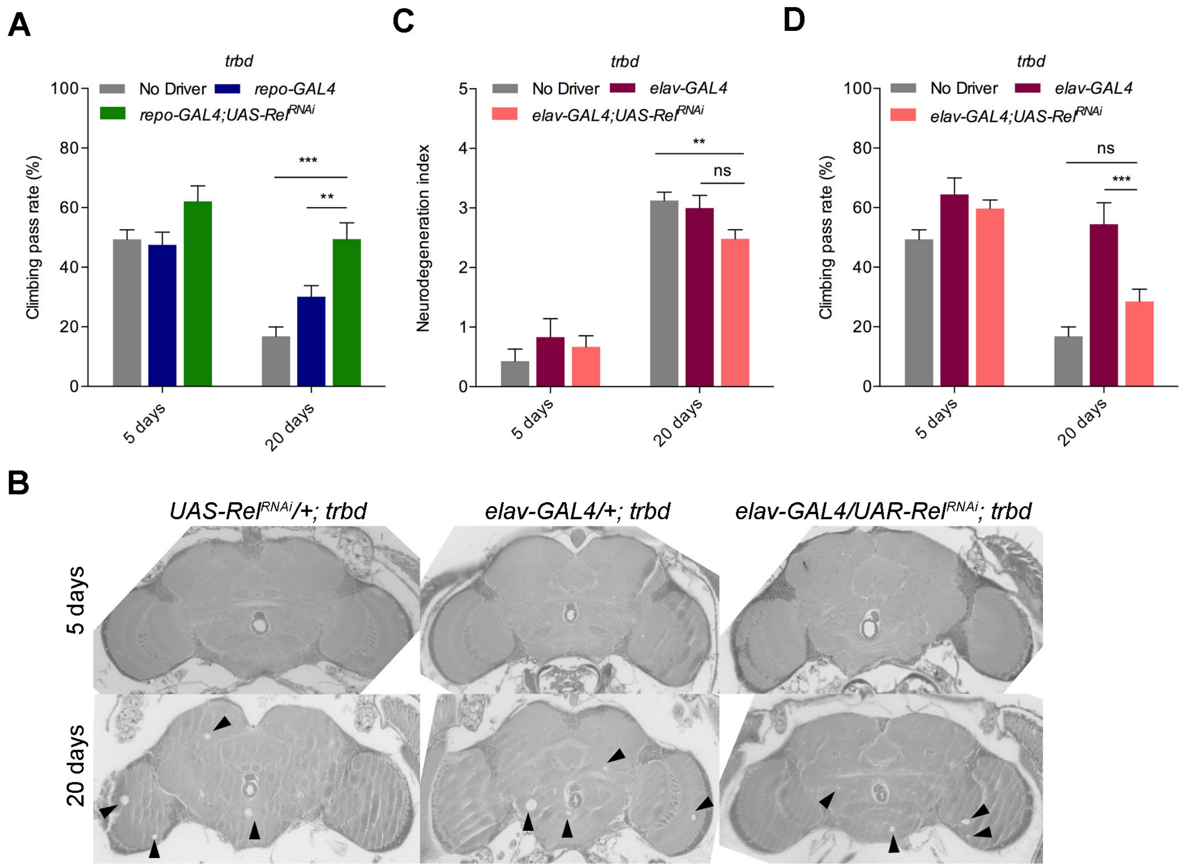

Figure S4 related to Figure 5.

Figure S4. Silencing *rel* expression in glia rescues *trbd* mutants. Locomotor activity was rescued by silencing *rel* in glia of *trbd* mutants **(A)**. In contrast, upon *rel* silencing in neurons neurodegeneration was only modestly reduced (**B** for sections, **C** for index) and locomotor activity was not rescued **(D)**. Values shown are mean ±SEM (* $p \leq 0.05$; ** $p \leq 0.01$; *** $p \leq 0.001$; ns, non-significant).

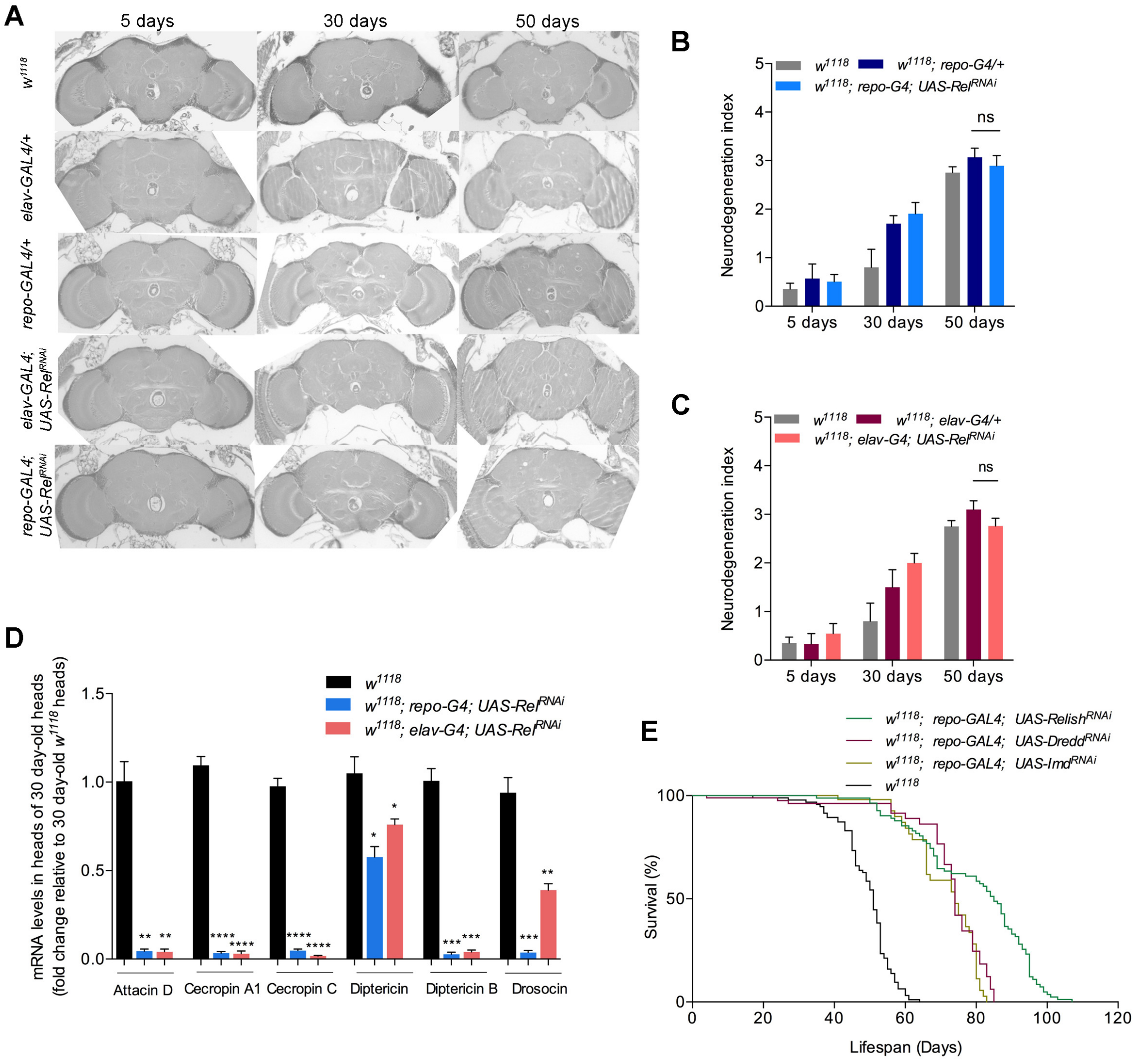

Figure S5 related to Figure 6.

Figure S5. Suppression of *rel* expression in brains of wild type flies. Silencing *rel* via RNAi in glia or neurons did not affect the age-dependent neurodegeneration in wild-type flies (**A** for sections; **B** and **C** for index). Both, neuronal and glial suppression of *rel* resulted in reduced AMP levels in heads of 30-day old w^{1118} flies **(D)**. **(E)** Lifespans of mated females, where *rel*, *Dredd* and *Imd* were silenced in glia. Values shown are mean ±SEM (* $p \leq 0.05$; ** $p \leq 0.01$; *** $p \leq 0.001$; **** $p \leq 0.0001$; ns, non-significant).

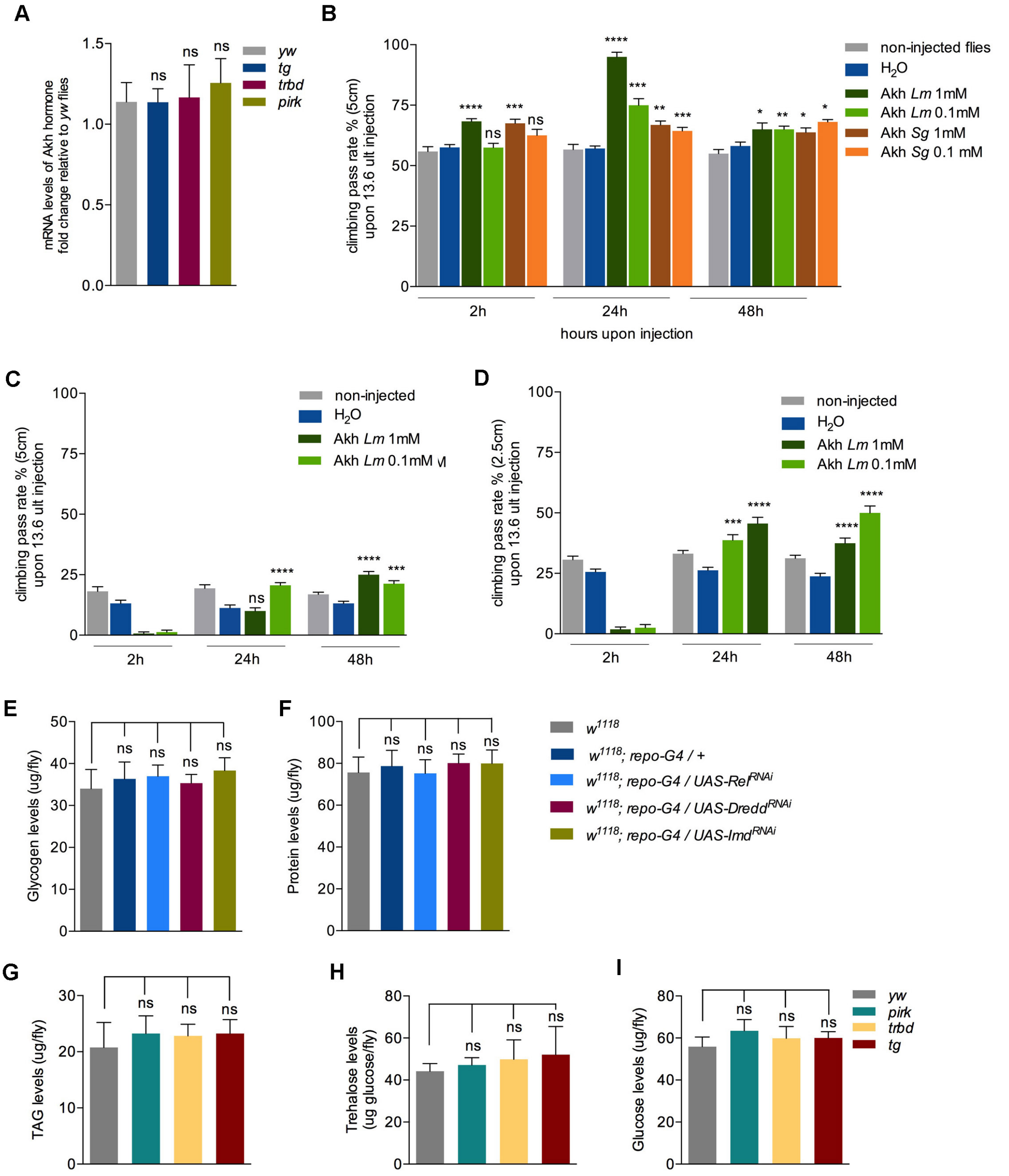

Figure S6 related to Figure 7.

Figure S6. Increased locomotor activity upon injection of Adipokinetic hormone. There was no change in endogenous *akh* gene expression levels in *tg, trbd* or *pirk* mutants (A). There was increased locomotor activity in 30-day old w^{1118} flies **(B)** and in 50-day old w^{1118} flies (**C**- 5cm pass height; **D** - 2.5 cm pass height) flies injected with 13.6 ml Adipokinetic Hormone from *Locusta migratoria* or *Schistocera gregaria*. Glycogen **(E)** and protein **(F)** levels of flies where *rel*, *Dredd* and *Imd* were silenced in glia were statistically indistinguishable from the control. This was also the case for TAGs (G) trehalose (H) and glucose (I) for mutants of *tg, trbd* and *pirk.* Values shown are mean ±SEM (* p ≤ 0.05; ** p ≤ 0.01; *** p ≤ 0.001; **** p≤ 0.0001; ns, non-significant).

Supplemental Experimental Pocedures

List of *Drosophila* strains

w^{1118} (BL #6326) and *yw67c23* (BL #6599), were used as controls and for the genetic background of all the other strains used in these studies; w^{1118}*,Dredd*^{EP1214} (BL #10456), *yw67c23,Dredd*^{B118} (BL #55712) w^{1118}*;;relish*^{E20} (BL #55714) w^{1118}*;;repo-GAL4* (BL #7415), *yw67c23;Tg*^{EY05203} (BL #15787), w^{1118}*;POSH*^{EP2248} (BL #17036), w^{1118}*;caspar* (BL #11373) were obtained from Bloomington Stock Center, w^{1118}*;CylD*^{f00814} was obtained from the Exelixis Collection and backcrossed into the w^{1118} background, w^{1118}*;UAS-relish*^{RNAi} (VDRC #49414) w^{1118}*;UAS-dredd*^{RNAi} (VDRC #28041) w^{1118}*;;UAS-imd*^{RNAi} (VDRC #1284) were obtained from the Vienna Drosophila RNAi Center; *yw67c23;;trabid* was obtained from Mariann Bienz (MRC Laboratory of Molecular Biology, Cambridge, United Kingdom) and backcrossed into the *yw67c23* background, *yw67c23;dRYBP*$^{\Delta 55}$*/CyO* was obtained from Ana Busturia (Centro de Biología Molecular "Severo Ochoa" CSIC-UAM, Madrid, Spain), w^{1118};*np1-GAL4* was obtained from Heinrich Jasper (Buck Institute for Research on Aging, Novato, CA, USA), w^{1118}*;elav-GAL4* was obtained from Efthimios Skoulakis (BSRC Alexander Fleming, Vari, Greece); w^{1118}*;PGRP-LB*$^{\Delta}$, w^{1118}*;UAS-AttA,* w^{1118}*;UAS-CecA,* w^{1118}*;UAS-Drc* were obtained from Bruno Lemaitre (Global Health Institute, EPFL, Lausanne, Switzerland). *Attacin-GFP* was obtained from David Wassarman (Laboratory of Genetics, University of Wisconsin-Madison).

Histology and neurodegeneration protocols

Briefly, flies were collected at 0-3 days of eclosion and aged at the respective temperatures (25°C or 29°C). Fly heads were severed and placed in fresh Carnoy's fixative (ethanol: chloroform: glacial acetic acid at the ratio 6:3:1) overnight at 4°C. Heads were then washed and placed in 70% ethanol and processed into paraffin using standard histological procedures. Embedded heads were sectioned at 5μm, and stained with hematoxylin and eosin. Images were taken under a Nikon light microscope (Nikon, Japan), equipped with a QImaging camera and images were generated using QImaging software (QImaging company, Canada) and processed with Photoshop CS5. Neurodegeneration is indicated by the appearance of vacuolar lesions in the brain neuropil. Six levels of neurodegeneration (0, 1, 2, 3, 4 and 5) are defined for quantification as previously described (Cao et al, 2013). The higher number indicates more severe neurodegeneration. For all samples, scoring of the neurodegeneration index was done blindly with respect to genotype. For sample size for each genotype see Supplemental Table.

Adipokinetic hormone injection assays

13.6 µl of Adipokinetic hormone II from *Locusta migratoria* (Abcam Biochemicals, UK) or from *Schistocera gregaria* (Abcam Biochemicals, UK) diluted in sterile water was injected in the thorax of adult flies using a micro-injector (Drummond Scientific, Nanoject II, USA) coupled to a fine glass needle. Control flies of same age were injected with 13.6 µl of sterile water.

Weight quantification

Total body weight of 10 individual flies was determined for each genotype. Each fly was then placed in an empty 2 ml eppendorf tube. The cap was left open and the flies were placed in a drying oven (70°C) with air blown through the chamber. The flies were left to dry for 24 hour and the dry mass was calculated. All measurements were made with a microbalance (Discovery, Ohaus, Switzerland).

Nutrient level assays

For all nutrient measurements, flies were frozen at −80 °C and homogenized in groups of five in 200 µl PBS + 0.05% Triton X-100. Samples were centrifuged at 1,000 × g for 2 mins to settle debris. All reactions were read with a micro-volume UV-Vis spectrophotometer (Nanodrop 1000, Thermo Scientific, UK).

For triacylglyceride (TAG) measurement, 10cm µl homogenate was placed in 200 µl triacylglyceride working reagent (Sigma-Aldrich, UK), spun down briefly in a micro-centrifuge following a 5-min incubation at 37°C and read at 540 nm compared with glycerol standards.

For glucose measurement, 10 µl homogenate was placed in 150 µl warmed Glucose reagent (Sigma-Aldrich, UK), spun down briefly in a micro-centrifuge following a 5-min incubation at 30°C and read at 340 nm compared with glucose standards

For protein measurement, 10 µl homogenate was placed in 200 µl of working reagent of PierceTM BCA Protein Assay Kit (Fischer Scientific, UK) following the manufacturer's protocol and read at 562 nm compared with BSA standards.

Food intake measurement

Flies were transferred to food 0.05% blue dye (FD&C Blue 80717 Sigma-Aldrich, UK), ten flies per sample. Flies were allowed to consume dyed food for 6 h and then were homogenized in 200 µL PBS + 0.05% Triton X-100 (PBST), centrifuged at 1,000 × g for 1 min to settle debris, and then 100 µL supernatant was read directly at 630 nm (reference wavelength of 670 nm) and compared with blue dye standards.

Behaviour assay
A negative geotaxis (climbing) assay was used to locomotor activity. The assay was performed in a room with controlled temperature of 21°C. For experiments presented in Figures 1, 3 and 5, groups of 10-30 flies from both sexes of each indicated genotype were collected at 0-3 days of eclosion and aged at 25°C. For experiments presented in Figure 4 and Figure S4, groups of 7-20 flies from both sexes of each indicated genotype were collected at 0-3 days of eclosion and aged at 29°C. For experiments presented in Figure S6, groups of 20 flies from both sexes of each indicated genotype were collected at 0-3 days eclosion, aged at 25°C and injected at the indicated age. In all experiments, flies were placed in the testing apparatus, consisting of two empty plastic vials taped together, and allowed to recover for 1 min. Next, flies were tapped to the bottom and let climb on the wall of the testing apparatus for 10 seconds. The number of flies that climbed over a 5cm line in 10 seconds was recorded. An additional series of tests using a 2.5cm line was followed for 50 day-old flies upon AKH hormone injections. The paradigm was repeated 5 times with 1 min of recovery between trials. Climbing pass rate was calculated by converting in percentage the average number of flies that climbed in all 5 trials. The number of independent replicates (n) and total number of flies tested for each genotype is presented in the Supplemental Table. Assays were performed on subsets of flies from vials used in the longevity experiments.

Single pair mating assays
One sexually naïve male was placed in a courtship chamber with one virgin receptive female and the pair was video recorded until the completion of mating. To attain a sexually experienced male, a 5-day old sexually naïve male was mated to a virgin female followed by a 30–45 min recuperation time at 25°C. The experienced male was then transferred to a new mating chamber with a virgin female and the pair was recorded until the completion of mating. Courtship index (CI) was estimated as the proportion of time a male spent courting (orientation, following, wing vibrations and abdomen bends) relative to the mating latency. Frequency and percent duration of wing extensions performed towards the female were calculated relative to the total male courting time. Abdomen bends included partial to full abdomen curvature when the male was oriented behind the female. Frequency of abdomen bends was estimated by recording the number of abdomen bends performed by the male and standardizing to courting time (N=20).

Immunohistochemistry
Fly brains were dissected in PBS1X and fixed in 4%PFA in PBS1X for 30 min, washed 3 successive times in PBS-Triton 0.03%, incubated 1h at room temperature in blocking solution (PBST, BSA 0.1%). Subsequently samples were incubated over night at 4°C with primary antibodies diluted in blocking solution. Samples were next washed 3 times with PBS-Triton 0.01% and incubated with secondary antibodies for 3h at room temperature. After 3 successive washes with PBS-Triton 0.01%, brains were mounted in Vectashield mounting medium and analyzed under confocal microscope Zeiss LSM510. Images were acquired with the Zeiss ZEN software and processed using Image J. The following primary antibodies were used: mouse anti-Repo (1:100) (#8D12, Developmental Studies Hybridoma Bank, University of Iowa, Iowa City, IA), rat anti-Elav (1:100) (#7E8A10, Developmental Studies Hybridoma Bank, University of Iowa, Iowa City, IA), and chicken anti-GFP (1:500), Life Technologies, Carlsbad, CA (reference #A10262). The following secondary antibodies were used at 1:500 dilutions: Alexa488-conjugated goat anti-chicken (Life Technologies, Carlsbad, CA, reference # A11039), Alexa633-conjugated goat anti-rat (Life Technologies, Carlsbad, CA, reference #A21094) and Alexa568-cojugated goat anti-mouse (Life Technologies, Carlsbad, CA, reference #A11021). DAPI (Sigma-Aldrich, St. Louis, MO) was added to the last PBS-Triton 0.01% wash prior mounting at the concentration of 1 mg/L.

Gene Expression Analysis – ddCT Method
Expression values were calculated using the DDCt method and normalized to rp49 expression levels. There are several methods of reporting real-time PCR data including absolute or relative expression levels. Absolute expression provides the exact copy number following transformation of the data via a standard curve. The data are typically presented as copy number per cell. In relative quantification, the real-time PCR data is presented relative to another gene often referred to as an internal control. Absolute quantification is required when a precise quantity of amplicon is desired, for example, calculation of viral load. The disadvantage of absolute quantification includes the increased effort to generate standard curves. Furthermore, it is often unnecessary to present data as absolute copy number and relative expression will suffice.

Several methods have been developed in order to present the relative gene expression. The efficiency correction method calculates the relative expression ratio from the real-time PCR efficiencies and the Real-time PCR data has been analyzed using the so-called sigmoidal curve fitting methods that fit the experimental data to an empirical equation and results in the prediction of the PCR efficiency and an estimate of the initial copy number of the amplicon.

Another method is the comparative ddCT method which makes several assumptions, including that the efficiency of the PCR is close to 1 and the PCR efficiency of the target gene is similar to the internal control gene.

There are advantages and disadvantages to each of the methods to analyze relative real-time PCR data. An advantage of the efficiency correction method is that the PCR efficiency of the target and internal control genes are included in the equation. The sigmoidal curve fitting models have the advantage that PCR efficiency does not need to be calculated by a separate experiment and is estimated during the analysis. Advantages of the comparative ddCT method include ease of use and the ability to present data as 'fold change' in expression

Disadvantages of the comparative CT method include the assumptions of PCR efficiency (alternatively the PCR must be further optimized) and the use of nonlinear regression analysis for the calculations. In our case, all of our primers' efficiencies were previously checked showing an efficiency of over 95%.

For more details please check the following references, Livak *et al,* 2001; Pfaffl 2001; Schmittgen *et al,* 2008.

Results are shown as Average ± SD of at least 3 independent biological samples.

List of primers
akh forw: TCCCAAGAGCGAAGTCCTCA
akh rev: CCAGAAAGAGCTGTGCCTGA
attacin A forw: CTCCTGCTGGAAAACATC
attacin A rev: GCTCGTTTGGATCTGACC
attacin B forw: GGGTAATATTTAACCGAAGT
attacin Brev: GTGCTAATCTCTGGTCATC
attacin C forw: CTGCACTGGACTACTCCCACATCA
attacin C rev: CGATCCTGCGACTGCCAAAGATTG
attacin D forw: AGTGGGGGTCACTAGGGTTC
attacin D rev: GTGGCGTTGAGGTTGAGATT
basket forw: CACCAACACTACACCGTCGA
basket rev: AAGCGGCGCATACTATTCCT
caspar forw: GAATCTTGTGGAGGCTCTAAGTC
caspar rev: GCACCAGGATAGGATGGGGA
caudal forw: CCATCGAAGCCGCCATACT
caudual rev: TTTGCCTGGTTGTGGTTGTG
cecropin A1 forw: CATTGGACAATCGGAAGCTGGGTG
cecropin A1 rev: TAATCATCGTGGTCAACCTCGGGC
cecropin A2 forw: ATTAGATAGTCATCGTGGTT
cecropin A2 rev: GTGTTGGTCAGCACACT
cecropin B forw: GAACGCATTGGTCAGCTAC
ceropin B rev: AGCGGTGGCTGCAACATT
cecropin C forw: TGTAAGCTAGTTTATTTCTATGG
cecropin C rev: GATGAGCCTTTAATGTCC
cyld forw: ATCGAGGTAGAAGACGAATCCA
cyld rev: GCATCTGTTGGCTGGTACAAAA
dif forw: GGAGCCGACAAGCAATATAATCC
dif rev: GTAGTTGCACACTTCGATGGT
diptericin forw: ACCGCAGTACCCACTCAATC
diptericin rev: GGTCCACACCTTCTGGTGAC
diptericin B forw: AGGATTCGATCTGAGCCTCAACGG
diptericin B rev: TGAAGGTATACACTCCACCGGCTC
dnr1 forw: CATTGTCAACCTGCCCAAC
dnr1 rev: GCGACAGACCTTCTCCAGAC
dredd forw: CAAAAGGTGGGCCTCTGCT
dredd rev: GTAGGTGGCATCCGAGTGGT
drosocin forw: GTTCACCATCGTTTTCC
drosocin rev: CCACACCCATGGCAAAAAC
drosocin forw (brain qPCR): CACCCATGGCAAAAACGC
drosocin rev (brain qPCR): TGAAGTTCACCATCGTTTTCCTG
drosomycin forw: AGTACTTGTTCGCCCTCTTCGCTG
drosomycin rev: CCTTGTATCTTCCGGACAGGCAGT
rybp forw: CATGTTGACACCTGGCTCCTG
rybp rev: CGAAGGTGATCGAGGAGAAC
dUSP36 forw: CAACACCTGCTACCTCAACTC
dUSP36 rev: CTGCTCCGAAACGAGCCAAT
Myd88 forw: ATCTGGAACACTTCCTGGGC

Myd88 rev: CCACGAGAGCAGTCTGTCG
PGRP-LB forw: CTTGTTTGTTTGTTTATTTTTGTG
PGRP-LB rev: CGGTAACCGTCGAGGC
PGRP-LC forw: TCCAATCGAAATCGGAAGAG
PGRP-LC rev: GGCGAAGATGTCTTTCCAAC
PGRP-LE forw: GATGCCGACCAAAATACCAG
PGRP-LE rev: GTCTTCGAAATGTGTCGGAG
PGRP-SC1a forw: AAGCGATCGTCAACTATTACAGC
PGRP-SC1a rev: GAGAGCCACTTTGGAAACCA
PGRP-SC1b forw: AGCTTCCTGGGCAACTACAA
PGRP-SC1b rev: GAGATCATGTTCGGCTCCAG
PGRP-SC2 forw: TGACCATCATCTCCAAGTCG
PGRP-SC2 rev: CAGCGAGGTCTTGCTCGT
pirk forw: CGATGACGAGTGCTCCAC
pirk rev: TGCTGCCCAGGTAGATCC
posh forw: CACACGTTAAACGACCTGTTG
posh rev: GCATCGCAACTTGTGCTGAC
relish forw: ACAGGACCGCATATCG
relish rev: GTGGGGTATTTCCGGC
rp49 forw: AAGAAGCGCACCAAGCACTTCATC
rp49 rev: TCTGTTGTCGATACCCTTGGGCTT
rp49 forw (brain qPCR): GACGCTTCAAGGGACAGTATCTG
rp49 rev (brain qPCR): AAACGCGGTTCTGCATGAG
tg forw: CATACGAGTCACTTCTATGCG
tg rev: CTTCAGCCGGAAGGGTTCTC
trbd forw: ATGTGAAACTTGCACCTACGAG
trbd rev: GCGACATTACGGCTACTTCG

Supplemental References

Livak K.J., and Schmittgen, T.D. 2001. Analysis of relative gene expression data using Real-Time Quantitative PCR and the $2^{-\Delta\Delta CT}$ method. Methods *25(4)*: 402-8.

Pfaffl M.W. 2001. A new mathematical model for relative quantification in real-time RT–PCR. Nucleic Acids Res *(29)9:* e45.

Schmittgen, T.D. and Livak K.J. 2008. Analyzing real-time PCR data by the comparative CT method. Nature Protocols *3(6)*: 1101-8.
